# Thermal runaway of large automotive Li-ion batteries

**DOI:** 10.1039/c8ra06458j

**Published:** 2018-11-30

**Authors:** Andrey W. Golubkov, René Planteu, Philipp Krohn, Bernhard Rasch, Bernhard Brunnsteiner, Alexander Thaler, Viktor Hacker

**Affiliations:** Kompetenzzentrum – Das Virtuelle Fahrzeug Forschungsgesellschaft mbH Inffeldgasse 21a A-8010 Graz Austria andrej.golubkov@alumni.tugraz.at; AVL List GmbH Hans-List Platz 1 8020 Graz Austria; Institute of Chemical Engineering and Environmental Technology, Graz University of Technology Inffeldgasse 25/C/II 8010 Graz Austria

## Abstract

Damaged or heavily over-heated Li-ion batteries in electric vehicles can transit into a thermal runaway reaction with further heat and gas release. The heat may cause a battery fire and fast gas release may damage the battery-pack casing. To characterise heat and gas release of large automotive Li-ion cells, a heavy duty test bench was developed and a test series was performed.

## Abstract 2

Damaged or heavily over-heated Li-ion batteries in electric vehicles can transit into a thermal runaway reaction with further heat and gas release.

## Graphical abstract

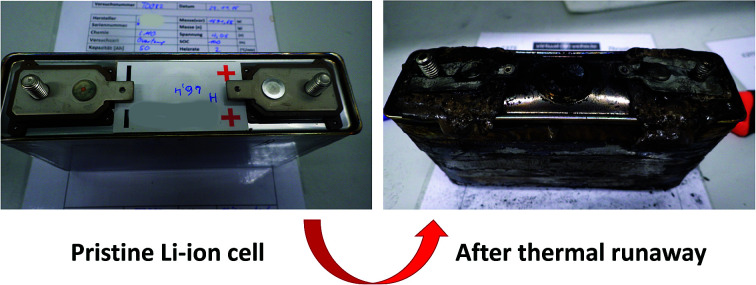

## 1 Introduction

**Fig. 1:**
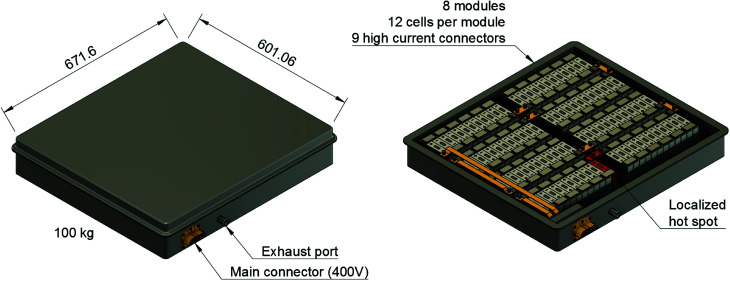
Drawing of a hypothetical PHEV battery-pack to demonstrate the safety issues: (left) including complete casing, (right) with upper part of the casing removed. A possible hot spot is shown in red. Dimensions in mm.

A typical application for a battery pack is a plug-in hybrid electric vehicle (PHEV): a PHEV with an electric range of 70 km needs a battery which can store 13 kW h of electric energy. Such a battery pack may consist of 96 large cells fixed inside the battery-pack casing as shown in Fig. 1. The battery pack is often fitted inside the available space in the luggage compartment of the car. Then, the only barrier between the passengers of the car and the Li-ion cells is the casing of the battery pack. The casing must protect the occupants from any gas or heat emission of the Li-ion cells.

PHEV batteries can be always energized, even when the battery pack is disconnected from the rest of the vehicle. The potential energy is a source of hazard and its uncontrolled release can easily cause battery fire.^1–7^ Examples of unwanted initial energy release are:

• Short circuits inside a cell.^8^

• Short circuits between cells, busbars and other metallic components.

• Failures inside the cell supervision circuits.

• Hot spots from damaged electric connectors.

All those failures can cause local heating of adjacent Li-ion cells. Charged cells are vulnerable to elevated temperatures because heated cell components can overcome chemical activation energy and decompose in exothermic chemical reactions. In the worst case, if heated to a critical temperature, the unwanted self heating rate of the cell becomes larger than the heat dissipation rate and the cell will transit into the so called thermal runaway. In case of charged Li-ion cells with high energy density, the thermal runaway is a fast, violent, self accelerating chemical reaction of the electrodes and the electrolyte which releases high amounts of heat and gas: cell temperatures up to 1000 °C and gas release of up to 25 mol kW^−1^ h^−1^ (600 L kW^−1^ h^−1^) were measured in previous work.^9^ The main components of the gas were CO_2_, CO and H_2_ making it burnable^10,11^ and toxic.^12–15^

In this work we address two safety topics for an automotive battery pack:

• The immediate risk of toxic gas intrusion from a vented cell into the passenger compartment and the countermeasures which consist of making a sturdy battery pack casing which can withstand some overpressure and including an appropriately sized exhaust port.

• The risk of thermal runaway propagation^16,17^ from cell to cell and the countermeasure of utilizing adjacent heat capacities, heat barriers and active cooling to keep the failure localised.^18,19^

To quantify those risks a test stand was built to measure the characteristic temperatures and gas emission rates of an automotive cell during thermal runaway.

This paper describes the tested cells, the test bench for thermal runaway experiments, and the methods for calculating the main thermal runaway parameters. The used methods to initiate thermal runaway of large Li-ion cells, to quantify the gas emission (amount, production rate, gas temperature) and to measure the cell-temperature are explained and an overview of the results from a test series and some details on exemplary tests are provided. After understanding the safety behaviour of the cells, we make first estimations for the design of the vent-gas exhaust of the battery-pack casing and we assess the risk of thermal runaway propagation.

## 2 Tested Li-ion cells

**Fig. 2:**
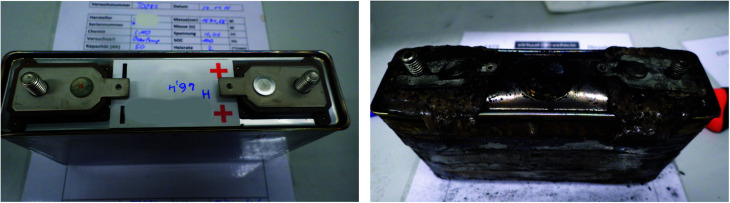
Tested cell before and after the thermal runaway experiment. On the fresh cell, the burst-plate of the cell is hidden below the white sticker. After a thermal runaway the cell is heavily damaged: the burst plate is open, the plastic insulation of the terminals is melted and the cell casing is deformed by the pressure inside the cell.

Large automotive cells designed for EV applications^20^ (Table 1) were tested. Each cell consists of a hermetically sealed prismatic casing made from stainless steel which contains the electrodes assembly. The positive and negative terminal and the rupture plate are integrated in the upper side of the casing (Fig. 2). The cells had been manufactured around the year 2009 and were stored until 2016. During this time their capacity decreased from 50 A h to 47 ± 2 A h due to ageing.

**Table 1:** Specifications of the tested Li-ion cells

Cathode	LiMn_2_O_4_ (LMO)
Anode	Graphite
Mean voltage	3.7 V
Nominal capacity	50 A h
Maximal current	300 A
Dimensions	113.5 mm × 43.8 mm × 171 mm
Mass	1.7 kg
Specific energy	109 W h kg^−1^

Compared to modern cell designs, those cells have low energy density. They use LMO cathodes whereas modern cells use mostly NMC and NCA cathodes. Test with new automotive cell-types with improved energy density (>200 W h kg^−1^) will be shown in follow-up publications.

## 3 Test stand for thermal runaway experiments

**Fig. 3:**
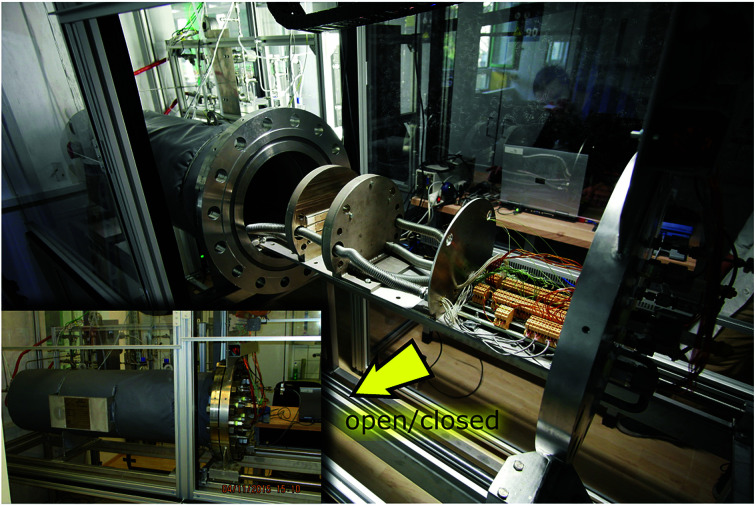
Reactor in opened and closed state.

The test stand for thermal runaway experiments was designed and built. The central component of the test stand is the heavy-duty gas-tight reactor with a volume of 121 l and a pressure rating of 40 bar. The reactor is limited to tests with cells and modules sized up to 1000 W h in nitrogen atmosphere. Test samples with higher energy content could possibly exceed the maximal pressure rating resulting in damaged test setup. Tests must be made in nitrogen atmosphere to avoid dealing with explosive gas mixtures. The main body of the reactor is fixed. The blind flange can be unscrewed from the main body and slid open along the rails until the attached cell holder stage is revealed outside of the reactor. Fig. 3 shows the reactor in opened and closed state. The blind flange also provides all electrical feedthroughs for the sensors, the electrical heating of the cell holder and the electrical connection to the cell.

**Fig. 4:**
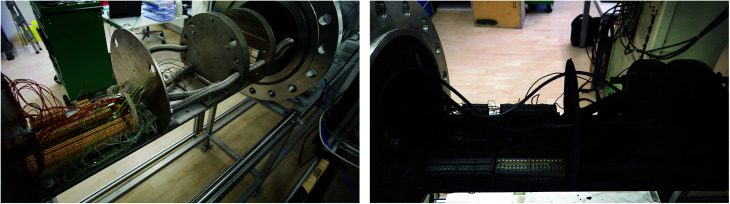
View of the inside of the reactor, before and after a couple of thermal runaway experiments. The inside of the reactor became coated with anode and cathode particles which were vented by the cells during the experiments.

**Fig. 5:**
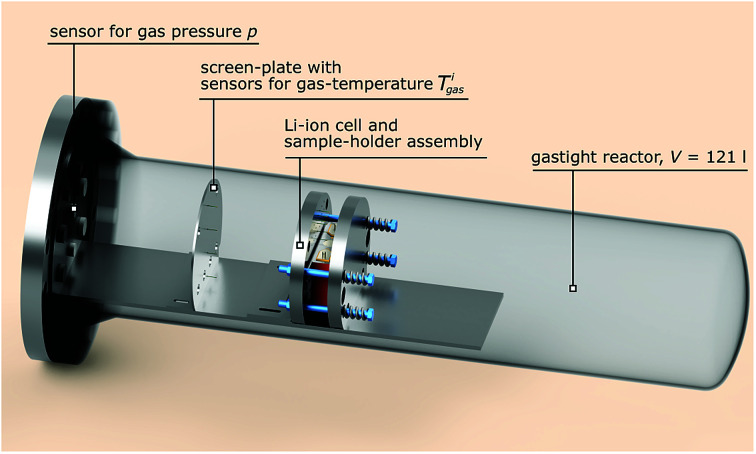
Simplified sketch of the reactor in closed state, wiring not shown.

The reactor contains the cell-holder for the Li-ion cell, a screen plate with gas-temperature sensors and heat resisting wiring (Fig. 5). All components inside the reactor must withstand some abuse by the violent thermal runaway reactions of the Li-ion cells (Fig. 4)

### 3.1 Cell holder

**Fig. 6:**
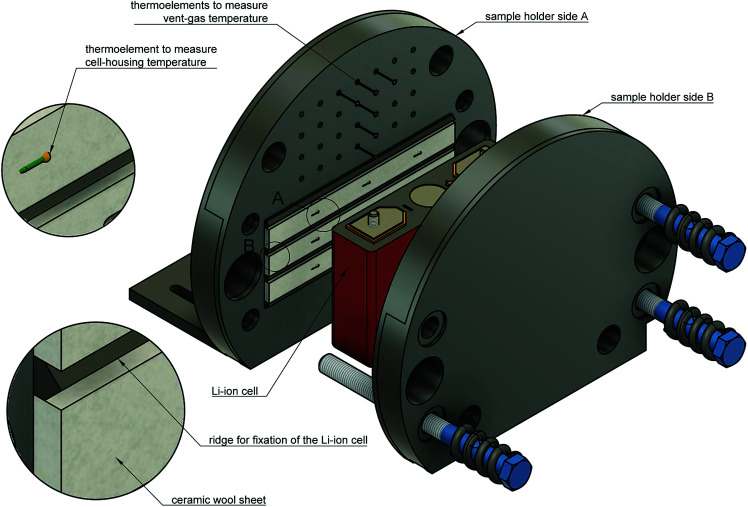
Exploded view of the cell holder and the Li-ion cell.

The Li-ion cell is fixed inside a custom made cell holder. The cell holder and the cell sample are placed into the reactor. The geometry of the cell holder is optimised to the cylindrical shape of the reactor (Fig. 6).

The functions of the cell holder are:

1. Applying mechanical pressure on the sides of the cell to simulate the mechanical situation in a battery module.

2. Heating of the cell.

3. Sensor fixture for the measurement of the cell casing temperatures.

4. Sensor fixture for the measurement of the vent gas temperature.

The two plates with the cell sandwiched in between are pressed together by metal springs. Each plate is equipped with internal heating cartridges (maximal heating power of 1200 W). The cell holder can heat with different heating rates depending on the settings of the power controller.

At cell temperatures above a critical temperature exothermic reactions inside the cell start and the cell begins to self heat. The objective is to detect the minimal temperature, at which self heating is evident. To increase the sensitivity of exothermic-event-detection the cell holder must not interfere with the self heating of the cell: the cell should perform the exothermic reactions as adiabatic as possible. To minimise the thermal coupling of the plates and the cell we put insulation sheets made from ceramic wool in between (heat conductivity *λ* = 0.1 W m^−1^ K^−1^, thickness *d* = 5 mm).

The plates also provide mechanical fixtures for temperature sensors. Each sample holder-plate has 9 positions for thermocouples. The tips of the thermocouples are positioned at equal distances along the cell-casing. They protrude through the insulation material and are pressed against the cell casing. Further thermocouples are placed in the space directly above the rupture-plate of the cell in order to measure the vent-gas temperature during the cell venting.

### 3.2 Screen plate

The screen plate is placed inside the reactor between the cell holder and the flange. It has two functions (1) mechanical protection of electrical wiring and connectors behind the screen and (2) fixture of four thermocouples at different heights to measure the mean gas temperature inside the reactor. The tips of the thermocouples are bare and uninsulated and they protrude through the screen plate towards the cell holder for 10 mm. This setup facilitates fast take up of temperature-change of the surrounding gas and minimizes the time delay of the temperature measurement.

### 3.3 Pressure sensor

Off-the-shelf pressure sensor (GEMS 3300B06B0A05E000) to measure gas pressure inside the reactor was used. The sensor is attached at the blind flange.

### 3.4 DAQ system

The DAQ system consists of standard modules in a cDAQ-9178 chassis from National Instruments. In this setup 30 channels for temperature measurements (k-type thermocouples), one channel for pressure measurement and one channel for cell voltage measurement with a sampling rate of 50 S s^−1^ for each channel were used.

In experiments for this work 18 thermocouples measured the cell surface temperature, 6 measured the vent-gas temperature near the burst-plate of the cell and 4 were attached to the screen plate to measure the gas temperature inside the reactor. The validity of the system can be checked by comparing measurements of the thermocouples to RTD sensors and by comparing the pressure signal to read-out of a manual manometer.

## 4 Methods and definitions

### 4.1 Initiation of thermal runaway

Thermal runaway of Li-ion cells can be triggered by localised or homogeneous heating and by overcharge. In the experiments we compare the thermal runaway initiated by four methods:

#### Heat ramp

Homogeneous heating of both large areas of the cell with constant temperature rate (both sample holder plates heat).

#### One-sided heating

Homogeneous heating of one single large area of the cell with constant temperature rate (only one sample-holder plate heats).

#### Stepwise heating

Homogeneous heating of both large areas of the cell with temperature steps (both sample-holder plates heat in steps).

#### Reactor heating

The whole reactor is heated from outside by the reactor heater.

### 4.2 Max cell temperature

The maximal cell case temperature *T*^max^_cell_ is the maximum recorded value of any temperature sensor that measures the cell case temperature. In most cases the maximal cell case temperature was recorded at the centres of the two large side-areas of the cells.

### 4.3 Critical cell temperature

The cell transits into a rapid thermal runaway, after its hottest surface location exceeds a critical temperature. Here we define the critical temperature *T*^crit^_cell_ in two steps: in the first step we look at the time span immediately before the main exothermic event and select the hottest temperature sensor. This is where the imminent rapid thermal runaway of the cell will originate. In the second step we define the critical temperature *T*^crit^_cell_ as the temperature when the temperature rate of the selected sensor *Ṫ*_cell_ exceeds 10 °C min^−1^.

### 4.4 Fail temperature

The fail temperature *T*_fail_ is the average cell surface temperature when the cell fails electrically: its voltage drops to zero volt.

### 4.5 Venting temperatures

The first and second venting temperature *T*_V1_ and *T*_V2_ are the average cell surface temperatures at which the (first) minor and the (second) major venting occurs.

### 4.6 Maximal vent-gas temperature

The maximal vent-gas temperature *T*^max^_V2_ is the maximum recorded temperature of the gas which was emitted by the cell through the rupture plate of the cell.

### 4.7 Temperature in the reactor

The temperature in the reactor *T*_reactor_ is the mean gas temperature in the reactor at the end of the experiment (several minutes after the thermal runaway event), when the gas temperatures come to an equilibrium. Gas species that are emitted by the cell condensate, when *T*_reactor_ is below their boiling point.

### 4.8 Amount of gas inside the reactor

During the thermal runaway of a cell, gas is released into the sealed reactor. To estimate the amount of produced vent gas and the vent gas release rate we calculate the overall amount of gas *n*(*t*) inside the reactor with the ideal gas equation:1
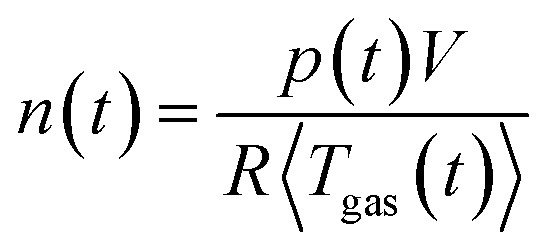
Here, the reactor volume *V* = 0.1208 m^3^, the gas constant *R* = 8.314 J mol^−1^ k^−1^, *p* (in Pa) is the gas pressure in the reactor and 〈*T*_gas_〉 (in K) is the average gas temperature in the reactor.

To measure the pressure is straightforward; a standard industry grade pressure sensor has sufficient resolution and fast step response. It is much more challenging to estimate the average gas temperature 〈*T*_gas_〉. The exact definition of 〈*T*_gas_〉 contains the 3D integral of the temperature over the volume *V* inside the reactor:2
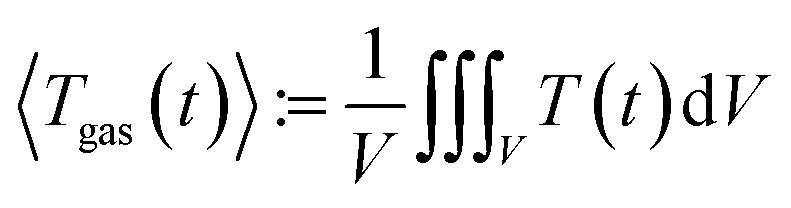


It is not possible measure the gas temperature at every point of space inside the reactor. Instead, we are limited to four temperature sensors which are fixed by the screen plate at four different heights (*Z*-direction) inside the reactor (Fig. 5). We approximate the volume-integral with the average of the sensors *T*^i^_gas_ (in °C) and apply a correction factor *c*_g_ and then convert from °C to K:3
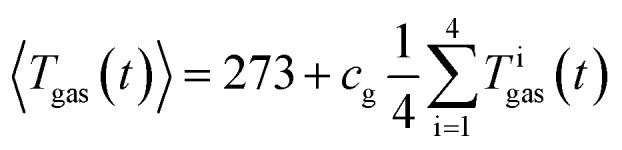


The factor *c*_g_ compensates the temperature deviations originating from convective flow between the heated sample holder and the not heated reactor (*vice versa* in case of experiment 10 and 11). The values of *c*_g_ typically range between 0.85 and 0.95.

The *c*_g_ was evaluated by looking at the value of *n* between the start of experiment and the first venting of the cell. At the beginning of the experiment the reactor and the cell are gas tight sealed. The heating ramp is started and when the temperature of the cell is high enough, its rupture disc burst and causes abrupt gas release into the reactor. The amount of gas *n* from the start of the heating ramp to the burst disc opening remains constant and corresponds to (1). The value of *c*_g_ is adjusted, so that the calculated *n* indeed stays constant between the start of experiment and first venting.

### 4.9 Amount of released gas

**Fig. 7:**
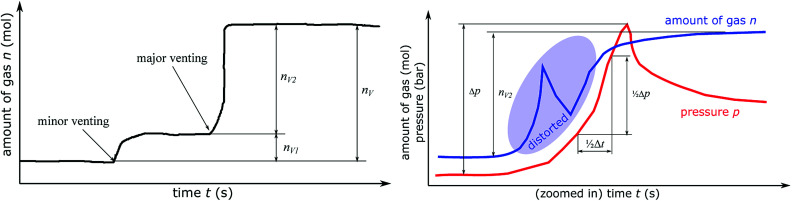
(left) Sketch of the amount of gas inside the reactor during a typical thermal ramp experiment. The cell can produce gas in two venting events: the first (minor) venting and the second (major) venting. (right) The method to calculate the characteristic venting rate *ṅ*_ch_. The amount of gas *n* can not be used directly because *n* is calculated from gas temperature measurement, which is distorted by violent gas flows during the main venting event. Instead, it is assumed, that 50% of *n*_V2_ is released during 1/2Δ*t* and 1/2Δ*t* is the timespan during which pressure rises by 1/2Δ*p*. The pressure based method can characterise high venting rates, because it is less affected by gas flows.

In the thermal ramp experiment the sample holder heats the cell and the cell releases gas in either only one (only major venting) or two subsequent events (minor venting followed by a major venting) (Fig. 7). The sum of the gas releases is the overall released vent-gas during the experiment:

4 *n*_V_ = *n*_V1_ + *n*_V2_

#### Minor gas release *n*_V1_

Increased cell temperature facilitates electrolyte decomposition into gaseous products. The build up of decomposition gas increases the internal cell pressure and causes deformations of the casing. High cell temperature, deformations and overpressure eventually cause the opening of the rupture disc. At the moment when the cell is no longer gas tight, it releases the surplus gas into the reactor. The gas release can be abrupt and the cell might cool down slightly (Joule–Thomson effect). The temperature of the surplus gas is below 220 °C.

#### Major gas release *n*_V2_

Either an internal short circuit or further temperature-increase and exothermic reactions start an accelerating chain reaction (thermal runaway), resulting in a major violent gas release. The temperature of the vent-gas is comparable to the maximum cell case temperature (>400 °C). The amount of gas in the major gas release is always higher than in the minor release (*n*_V2_ > *n*_V1_).

### 4.10 Venting-rate of the gas

The venting sub system of the battery pack casing must ensure, that the gas is safely transported to the outside of the vehicle. Therefore, the speed of the gas release during the major venting *n*_V2_ is crucial. We define the characteristic venting-rate *ṅ*_ch_ as the ‘speed’ of gas release (in mol s^−1^). This so called venting rate is the most important parameter needed to calculate the dimensions of the vent gas pipe of the battery pack.

A simple algorithm calculates the characteristic venting-rate by estimating the minimum time which is needed to vent 50% of *n*_V2_. By choosing 50% of pressure rise, the algorithm automatically selects the main phase of fast gas release in the middle of the venting event and it excludes the slow onset and the final trailing-off of the gas emission.

The algorithm mainly depends on the pressure signal because pressure is less prone to be obfuscated even by violent venting (Fig. 7). The pressure has the value *p*_0_ before the major venting, during major venting it reaches a maximum *p*_max_ and then it slowly decreases to *p*_1_ as the released hot vent-gas cools down. We define Δ*p* = *p*_max_ – *p*_0_ as the maximal pressure change (Fig. 7). In the next step the algorithm scans the whole time-series of the *p*_k_-signal. Here k is the index of the time series running from 1 to *e.g.* 300 000 for an experiment duration of 6000 s and a time resolution of *r* = 0.02 s. The algorithm starts at every *p*_k_ and increases the index *l* until

5 *p*_k+l_ − *p*_k_ > 1/2Δ*p*

The value of *l* is proportional to the time which is needed to vent 50% of gas (1/2Δ*t* = *rl*). The value of *l* is stored in *D*_k_ = *l*. After calculating *D*_k_ for every k the minimum of *D*_k_ is used to calculate the characteristic venting rate *ṅ*_ch_:6
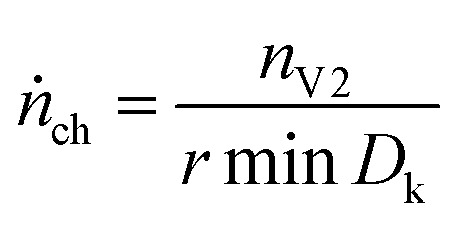


In other words, the algorithm scans the whole length of the experiment and selects the time-span where the pressure changes by 1/2Δ*p* in a minimum amount of time.

## 5 Results

### 5.1 Overview

**Fig. 8:**
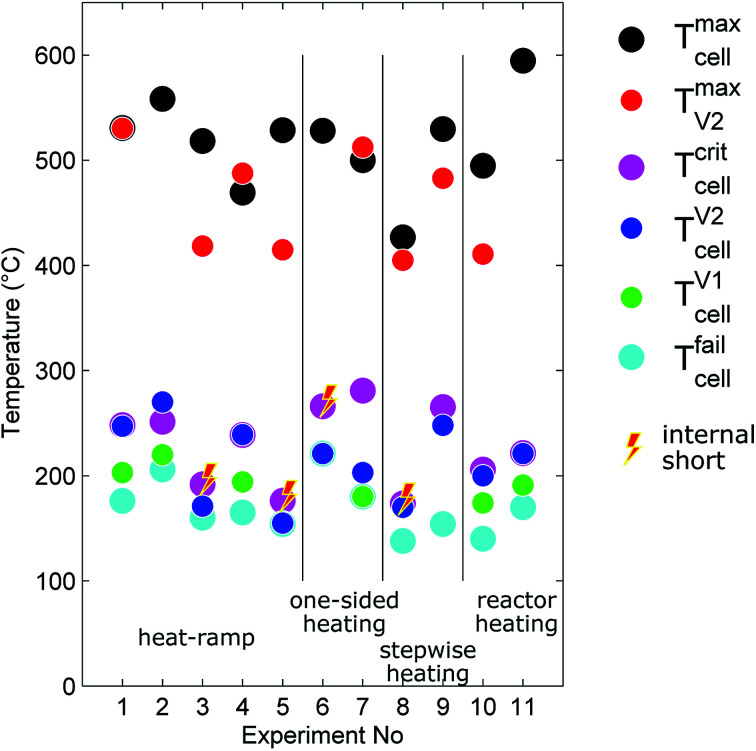
Characteristic temperatures associated with the events of cell failure (voltage drop), first and second gas release, reaching the critical temperature, reaching the maximal cell temperature during thermal runaway and the maximum vent-gas temperature.

**Fig. 9:**
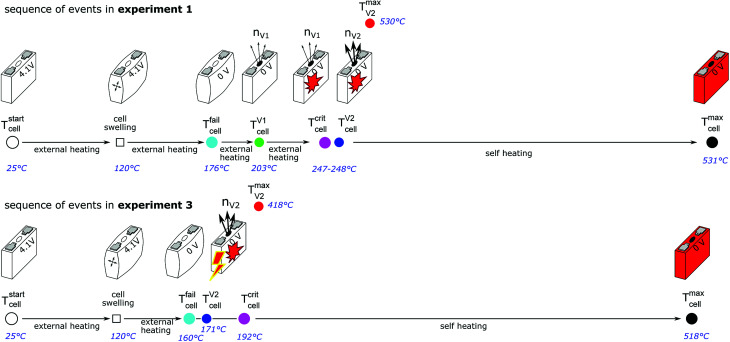
Sketch of the sequence of events as the temperature of the cell increases for experiment 1 and 3. In experiment 1 cell case swelling occurs at 120 °C, the cell fails at 176 °C, the burst disc opens and releases the overpressure from the cell into the reactor and some cell swelling is reversed at 203 °C then increasingly exothermic chemical reactions slowly evolve into a thermal runaway with a critical temperature of 248 °C and second major venting at 247 °C. The cell casing reaches a maximum temperature of 531 °C. In experiment 3 the cell also starts to swell at 120 °C and the cell fails at 160 °C. Then – at an average cell temperature of 170 °C and the hottest cell case sensor showing 192 °C – an internal short circuit triggers a sudden heat release which immediately causes venting and thermal runaway. The cell casing reaches a maximum temperature of 518 °C.

The outcome of thermal-runaway experiments with different heating methods is compared: five cells were heated by a heat ramp, two cells by one-sided heating, two cells by stepwise heating and two cells by heating of the whole reactor. All experiments were done in nitrogen atmosphere. The results from the eleven experiments are compiled in Table 2 and characteristic temperatures are compared in Fig. 8. Unfortunately, the cells showed different event sequences (Fig. 9) during repeated experiments. We identified three types of event sequences and categorised them in three groups:

**Table 2:** Results of all experiments. “–” means that the first (minor) venting did not take place. “n.a.” means not available, because associated temperature sensors failed or – in case of experiment 4 – because we forgot to measure the cell mass after the experiment. Here type is the initiation method to start the thermal runaway, *V*^before^_cell_ is the cell voltage before the experiment is started, group is the category into which the experiment result was classified, *T*^crit^_cell_ is the temperature at which the hottest cell case sensor exceeds 10° C min^−1^, *T*^fail^_cell_ is the average cell case temperature at which the cell voltage drops to zero, *T*^V1^_cell_ and *T*^V2^_cell_ are the average cell case temperatures at which the first and second gas release occurs, *T*^max^_cell_ is the maximal cell case temperature during the thermal runaway, *T*^max^_V2_ is the maximum vent-gas temperature, *n*_V1_ and *n*_V2_ are the amounts of released gas during first and second venting, *n*_*V*_ is the overall released amount of gas, *T*_reactor_ is the reactor temperature when *n*_V_ is measured, *ṅ*^ch^_V2_ is the characteristic venting rate during the main venting event, *M*^before^_cell_ and *M*^after^_cell_ are the cell masses before and after the experiment

No	Type	*V* ^before^_cell_ V	Group	*T* ^crit^_cell_ °C	*T* ^fail^_cell_ °C	*T* ^V1^_cell_ °C	*T* ^V2^_cell_ °C	*T* ^max^_cell_ °C	*T* ^max^_V2_ °C	*n*_V1_ mol	*n*_V2_ mol	*n*_V_ mol	*T*_reactor_ °C	*ṅ* ^ch^_V2_ mol s^−1^	*M* ^before^_cell_ g	*M* ^after^_cell_ g
1	Heat ramp	4.08	A2	248	176	203	247	531	530	0.4	2.8	3.2	61	0.9	1693	1092
2	Heat ramp	4.08	A2	251	206	220	270	558	n.a.	0.3	2.7	2.9	70	0.9	1695	1074
3	Heat ramp	4.06	B1	192	160	—	171	518	418	—	3.1	3.1	55	0.7	1695	1119
4	Heat ramp	4.06	A2	239	165	194	239	469	488	0.3	2.7	3.1	62	1.4	1691	n.a.
5	Heat ramp	4.10	B1	176	154	—	155	529	415	—	4.6	4.6	53	0.4	1638	1101
6	One-sided heating	4.08	B1	266	221	—	221	528	n.a.	—	3.2	3.2	58	0.6	1696	1156
7	One-sided heating	4.04	A2	281	180	180	203	500	512	0.5	2.8	3.3	43	0.6	1693	1177
8	Stepwise heating	4.08	B2	173	138	167	172	427	405	0.4	2.9	3.4	73	0.7	1696	1143
9	Stepwise heating	4.10	A2	265	154	210	248	530	483	0.3	3.3	3.6	69	0.7	1637	1063
10	Reactor heating	4.08	A2	206	140	174	200	495	411	1.1	4.5	5.6	249	1.3	1695	1115
11	Reactor heating	4.08	A2	222	155	191	221	595	n.a.	1.6	4.4	5.9	253	1.4	1696	1118

#### Group A2

Most experiments showed both venting events (minor and major) and a forerunning temperature increase immediately before the main exothermic reaction.

#### Group B1

In three experiments (experiment no. 3, 5, 6) the cell vented only once (only major venting during the main exothermic thermal runaway reaction) and showed no forerunning temperature increase.

#### Group B2

In one experiment (no. 8) we observed two venting events (as in A2) but no forerunning temperature increase.

The overall results show, that some thermal runaway characteristics depend and some do not depend on the heating method or group. The voltage of the cells broke down to 0 V at *T*^fail^_cell_ = 170 ± 30 °C for all groups and heating methods. Temperatures of the venting events showed more dependence on the group then on the heating method. In the group A2 the (first) minor venting occurred at an average cell casing temperature of *T*^V1^_cell_ = 200 ± 20 °C and the (second) major venting and thermal runaway reaction occurred at *T*^V2^_cell_ = 230 ± 30 °C. In the group B1, with only the major venting, venting occurred simultaneously with their thermal-runaway reaction at cell temperatures *T*^V2^_cell_ = 182 ± 30 °C. We suspect that – in contrast to group A2 – in group B1 the mechanical stress from cell swelling caused an internal short circuit, which, in turn, caused a very fast transition to a full thermal runaway. In other words, we suspect that in group B1 the minor venting, short circuit, thermal runaway and the major venting happened almost simultaneously so that the venting could not be resolved into a minor and major part.

**Fig. 10:**
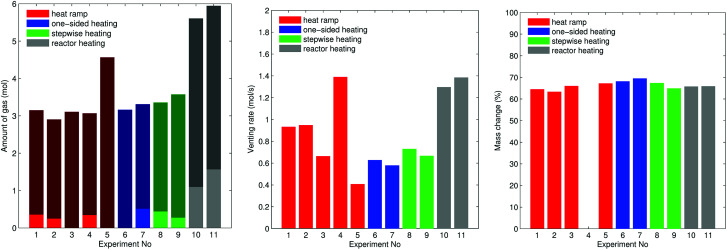
(left) Gas emission. Each bar shows the minor venting *n*_V1_ (if present) and the major venting *n*_V2_ on top. (center) Rate of gas emission *ṅ*^ch^_V2_. (right) Mass of the cell after the thermal runaway experiment compared to mass of the fresh cell.

The overall measured amount of released gas mainly depended on the reactor temperature (Fig. 10). In the first nine experiments (except for experiment no. 5.) with unheated reactor we measured *n*_V_ = 3.2 ± 0.2 mol. In experiment no. 5 the released amount of gas exceeded the average by 40%. Unfortunately we could not come up with any explanation for this outlier. In the experiments 10 and 11 the whole reactor was heated and a reactor temperature of 250 °C prevented condensation of electrolyte vapour. The additional gaseous electrolyte increased the amount of gas by 80% to *n*_V_ = 5.77 ± 0.2 mol.

Minor venting was only observed in the groups A2 and B2. In the experiments with unheated reactor minor venting contributed *n*_V1_ = 0.4 ± 0.1 mol to the overall amount of gas. In the last two experiments, with heated reactor, minor venting contributed by a higher amount of gas *n*_V1_ = 1.3 ± 0.3 mol because released electrolyte vapour stayed in gas phase.

The accumulation rate of gas in the reactor showed a high variation and no clear dependency on heating method (Fig. 10). In the first nine experiments, with unheated reactor, therefore not counting the electrolyte vapour, the cells released *ṅ*^ch^_V2_ = 0.8 ± 0.3 mol s^−1^. In the last two experiments, with heated reactor and therefore including the electrolyte vapour the cells released *ṅ*^ch^_V2_ = 1.3 mol s^−1^.

The maximum cell temperatures and maximum vent gas temperatures showed no apparent dependency on the heating method or group. We measured *T*^max^_cell_ = 520 ± 40 °C and *T*^max^_V2_ = 460 ± 50 °C.

The cells lost significant amount of mass during the thermal runway (Fig. 10). The mass of the cells decreased from *M*^before^_cell_ = 1680 ± 20 g to *M*^after^_cell_ = 1120 ± 40 g independent of group or heating method.

### 5.2 Heat ramp (group A2)

**Fig. 11:**
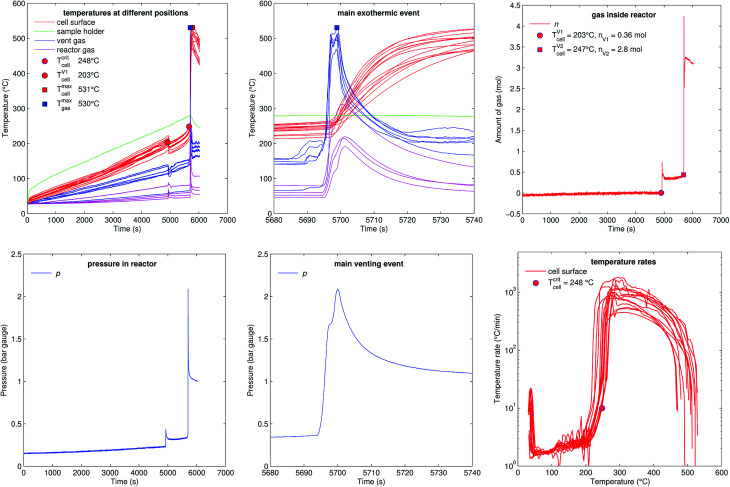
Experiment no. 1 (heat ramp). (left) Temperatures and gas pressure during the whole duration of the experiment and (centre) during the main exothermic event. (right) Amount of released gas into the reactor and temperature rates of the cell case sensors.

Fig. 11 shows the experiment no. 1. The cell was heated with a heating ramp of 2.4 °C s^−1^. The first minor venting occurred at 5000 s and the second main venting at 5700 s.

The combination of gas build up in the cell and the minor venting caused two effects which produced a measurable temperature drop of the cell case sensors at 5000 s. First effect: the Joule–Thomson effect causes some cooling of the cell and of the vent-gas temperature-sensors as the gas expands from the cell into the reactor. Second effect: the cell case swelling effect, this is more intricate. During heating above 120 °C gas builds up inside the cell and causes a bulging of the cell casing. The bulging forces the temperature sensors on the cell casing towards the heating side plates of the sample holder. At the same time gas inside the cell builds up an insulating gas layer between the active material (jelly roll) and the heated casing of the cell. The measured temperature increases, because the place which the sensors measure is shifted towards the hot cell holder plates and away from the cooler active material in the cell. Then, during minor venting, the built-up gas is released from the cell, the bulging of the cell casing is reversed, the cell casing with the attached temperature sensors comes in thermal contact with the cooler active material inside the cell and the temperature sensors measure a sudden temperature drop. (In other words, imagine a frozen ice cream inside an inflated air balloon with temperature sensors attached to the outer balloon shell. As long as there is air inside the balloon, the temperature sensors would measure the ambient temperature, but when gas is released they would measure the temperature of the cooler melting ice.)

Note that the duration of the major gas release – and also the duration of the thermal runaway reaction which causes the major gas release – at 5700 s was very short (2 s). The cell casing reached the maximal temperature 40 s after the major gas release and the completion of the thermal runaway reaction inside the cell. This delay of temperature response is caused by thermal masses and finite heat conductivity from inside the cell to the sensors on the outside of the cell (high Biot number). Therefore, we believe that the gas release duration is a much better indicator to judge the duration of the main thermal runaway reaction than the outside cell case temperature.

The hot gases (530 °C) from the major venting caused a maximal pressure of 2 bar in the reactor. Then the gas cooled down (to 61 °C) and partially condensed and the pressure dropped to 1 bar.

### 5.3 Heat ramp (group B1)

**Fig. 12:**
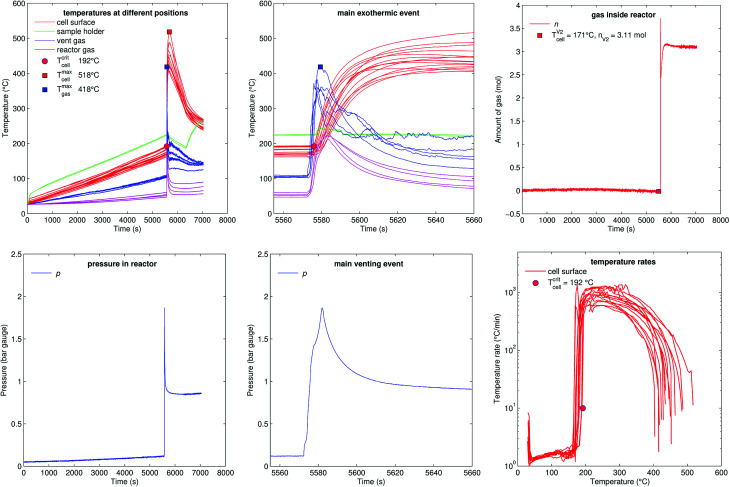
Experiment no. 3 (heat ramp), with an internal short circuit occurring at 171 °C. (left) Temperatures and gas pressure during the whole duration of the experiment and (centre) during the main exothermic event. (right) Amount of released gas into the reactor and temperature rates of the cell case sensors.

Fig. 12 shows the experiment no. 3. The setup was the same as in the experiment no. 1: the cell was heated from both sides with a heat ramp. In contrast to experiment no. 1 (classified to group A2) the cell showed a different sequence of events, classified as group B1: this time we could observe only one venting and a simultaneous thermal event at an average cell temperature of only 171 °C at 5572 s. Another difference was that the event was not preceded by any detectable self heating: the main event happened spontaneously without any warning (compare temperature plots in Fig. 11 and 12) and resulted in a maximum temperature of 518 °C. The cell in experiment no. 5 showed similar behaviour, with the main (and only) event at 155 °C.

We conclude that the spontaneous thermal runaway event was caused by an internal short circuit inside a cell. The short circuit occurred before the minor venting would normally happen and therefore the cell experienced only one major venting during the main exothermic reaction.

### 5.4 One-sided heating (group A2)

**Fig. 13:**
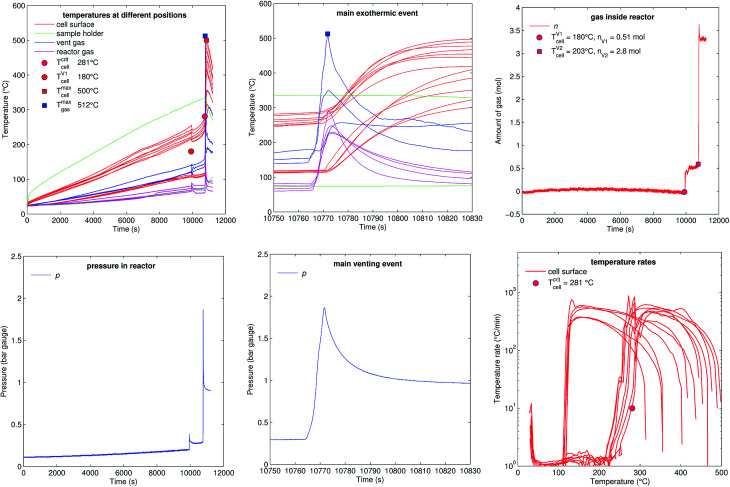
Experiment no. 7 (one sided heating). (left) Temperatures and gas pressure during the whole duration of the experiment and (centre) during the main exothermic event. (right) Amount of released gas into the reactor and temperature rates of the cell case sensors. Here the one-sided heating method created a huge temperature difference between the heated and the non-heated surface of the cell. This divided the cell case temperature sensors into two groups: ones that measured the heated side of the cell and ones that measured the cooler side. Sensors of both groups reached above 400 °C during the thermal runaway of the cell. Both groups are also clearly seen in the rate plot. The thermal runaway started, when the hottest sensor on the heated side of the cell reached 281 °C. The thermal runaway propagated throughout the cell from the hot side to the cooler side. After it reached the cooler side it caused a steep temperature rate increase in the sensors which were only at 120 °C.

Fig. 13 shows the experiment no. 7. Here the cell was heated from one side and the other side stayed unheated by the sample holder. This created a huge temperature gradient through the cell. At 10 000 s the cell vented for the first time and at 10 770 s the main exothermic event occurred. At this point the heated side of the cell was at 270 °C and the unheated side was at 120 °C. The thermal runaway caused cell temperature increase to a maximum of 500 °C.

### 5.5 Stepwise heating (group B2)

**Fig. 14:**
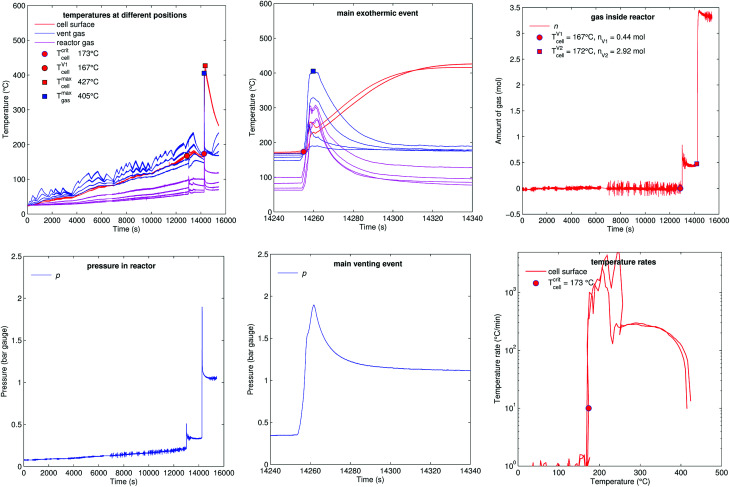
Experiment no. 8 (stepwise heating), unfortunately only two cell temperature sensors remained intact in this experiment. (left) Temperatures and gas pressure during the whole duration of the experiment and (centre) during the main exothermic event. (right) Amount of released gas into the reactor and temperature rates of the cell case sensors.

Fig. 14 shows the experiment no. 8. The cell was heated from both sides with subsequent temperature steps. In this experiment only two thermocouples could measure the cell case temperature. (The actual motivation for this experiment was to do Electrochemical Impedance Spectroscopy (EIS) measurements at different temperatures. The temperature steps look sporadic, because they were set manually during the experiment. EIS results could not be included in this paper.)

The cell showed the minor venting events at 13 000 s (releasing 0.4 mol), it was heated further to 180 °C then the cell was let to cool down to 167 °C. Surprisingly – after cooling down from 180 °C to 167 °C – a short circuit occurred and caused a thermal runaway at 14 250 s. The cell released additional gas during its major venting (2.9 mol, 405 °C) and the cell reached a maximal temperature of 427 °C.

### 5.6 Reactor heating (A2)

**Fig. 15:**
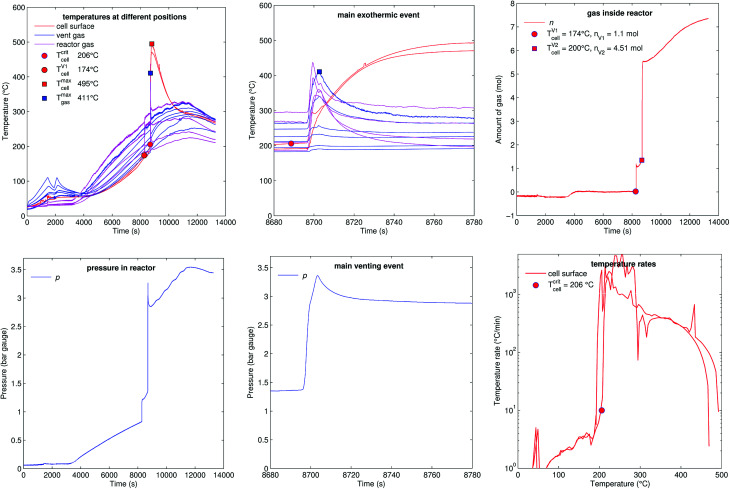
Experiment no. 10 (reactor heating), unfortunately only two cell temperature sensors remained intact in this experiment. (left) Temperatures and gas pressure during the whole duration of the experiment and (centre) during the main exothermic event. (right) Amount of released gas into the reactor and temperature rates of the cell case sensors.

Fig. 15 shows the experiment no. 10. Here not the sample holder, but the whole reactor was heated. The reactor reached a temperature above 200 °C when the cell went into thermal runaway. The main difference to the previous experiments was, that more of the released material stayed in the gas phase, because the hot walls of the reactor prevented condensation inside the vessel. After the minor venting at *t* = 8280 s the reactor contained *n*_V1_ = 1.1 mol of gas instead of 0.25–0.51 mol as in previous experiments. The additional amount of gas was likely composed of electrolyte-solvent vapours which stayed in gas phase because of high reactor temperature. A possible candidate for the additional electrolyte vapour could be ethyl methyl carbonate^21^ (EMC) with a molecular mass 104 g mol^−1^ and a boiling point of 110 °C. An additional amount of 0.59–0.85 mol of EMC vapour has a mass of 61–88 g which corresponds to about 5% of the cell mass.

The minor venting was followed by the main thermal event and major gas release at *t* = 8700 s: the cell reached a surface temperature of *T*^max^_cell_ = 495 °C and released additional *n*_V2_ = 4.51 mol of gas. The amount of gas *n* continued to increase after the major venting (from 5.5 mol to 7.3 mol), probably due to further thermal decomposition of the remaining solvent components on the hot reactor walls. The reactor heating was switched off at 11 500 s.

The calculated venting rate of the major event was higher than in most previous experiments *ṅ*^ch^_V2_ = 1.3 mol s^−1^. The increase in venting rate was not caused by a higher reaction rate of the cell, but by electrolyte vapour which contributed to a higher value of *n*_V2_.

## 6 Discussion

How are the results useful for designing safe battery packs? A robust battery pack should not allow propagation of thermal runaway from cell to cell and it needs safe vent-gas management.

### 6.1 Thermal runaway propagation

First we focus on the thermal runaway propagation: a chain reaction which occurs when a failed cell heats its adjacent cells to a point, where they transit into thermal runaway as well, they in turn infecting the next row of adjacent cells, ultimately ending in a full battery fire. The propagation progresses by heat transfer from one cell to the next. For stacked metal-can cells (arranged as in Fig. 1) the main thermal interfaces for heat exchange are the large areas on the sides of the cells.

We have shown that thermal runaway of the initial cell can be caused by heating or a combination of heating and internal short circuit. Without internal short circuit, if the cell is heated uniformly, the cell will transit into rapid thermal runaway when its hottest point exceeds 246 °C. In a subsequent thermal runaway reaction the first failed cell may reach a temperature of up to 595 °C. The failed cell will then transfer the majority of the produced heat to one or two of its adjacent cells: each adjacent cell will be heated from one side.

In our experiments we compared uniform and one sided heating. One sided heating creates a huge temperature gradient from heated to non-heated cell side. In our experiments non-uniform heated cells transited into rapid thermal runaway when their hottest point exceeded 281 °C (while the opposite site was only at 120 °C). This is in contrast to uniform heating, where the critical temperature is already at 246 °C.

Such results can be used to develop and to validate thermal runaway models. Such models can further be used to determine if – in a certain scenario – thermal runaway propagation is possible or not. For example; the outcome of the propagation study may depend on the position of the failed cell in the cell stack.

In a first scenario: consider that the failed cell sits in the middle of the cell stack. For some reason it goes into thermal runaway and instantly reaches 595 °C, while the rest of the cells stays at ambient temperature at 25 °C. Next, the major amount of heat will be transported from the failed cell to its left and its right neighbour. If local temperatures inside the neighbours would reach some critical temperature, they would transit into thermal runaway as well, and the fault propagation would start.

In a second scenario: consider that the failed cell is the outermost cell in a cell stack. In contrast to the first scenario the failed cell has only one adjacent cell with which it will share most of the generated heat. If no other heat sinks are provided then propagation is more likely to happen then in the first scenario.

In general, to prevent the propagation, the heat from the failed cell must be distributed to as many cells (or other components with thermal capacities) as possible, while keeping the local temperatures of all non failed cells below their critical temperature. If the recipient of all heat is only one adjacent cell, propagation is very likely. With several heat recipients, propagation is less likely.

### 6.2 Vent gas management

A robust battery pack also needs a vent-gas management which consist of a battery pack enclosure which can withstand some overpressure and a venting duct which can guide the vent-gas to the outside. The main parameters for the design of those venting elements are the gas release rate, gas temperature and the gas composition. The gas temperature and release rate were measured in our experiments and the gas composition can be estimated from our previous experiments.^9,12^

We measured the gas release rate inside unheated reactor and compared it to gas release in a heated reactor. Here the reactor simulated the environment inside a battery pack. The first failed cell would release gas into a pack with normal working temperature, and some gas components would condensate, before they exit through the venting port. If propagation occurs, the next cell would vent inside an already preheated pack, fewer gas would condensate inside the pack, and more gas would exit though the venting port. If propagation can not be excluded, venting tests should be done in a heated reactor. Our experiments showed a trend of higher venting rate for a heated reactor, but, unfortunately, also a high variation from experiment to experiment (Fig. 10).

For a first exemplary specification of the venting port we assume an isentropic flow of the vent-gas.^22^ The gas is released by the failed cell, then it flows inside the battery pack and exits through the venting port to the outside of the battery pack. We assume that the main obstacle to be the venting port of the battery pack with its limiting cross section *A*.

For a simple estimation we further assume that the vent-gas consists of equal mole parts of H_2_, CO_2_ and CO. The gas has a mean mol mass *M*_s_ = 0.02467 kg mol^−1^ and the mean isentropic expansion factor *γ* = 1.32 at a temperature of *T*^V2^_max_ = 800 K (Table 3). We further assume that the pressure inside the battery pack is *p*_0_ = 120 kPa and the ambient pressure is *p*_*t*_ = 100 kPa, meaning *p*_*t*_ − *p*_0_ = 200 mbar of overpressure caused by the failed cell during the venting event.

**Table 3:** Characteristics of typical vent-gas components at 800 K. The average is valid when the gas consists to 1/3 of each component. Retrieved from NIST Chemistry WebBook^23^

	*C*_p_ (J mol^−1^ K^−1^)	*C*_v_ (J mol^−1^ K^−1^)	*γ*	*M* (g mol^−1^)
H_2_	29.65	21.34	1.39	2.02
CO	29.81	21.48	1.39	28.01
CO_2_	51.46	43.13	1.19	44.00
Average			1.32	24.67

The flow factor *Ψ* is given by:7
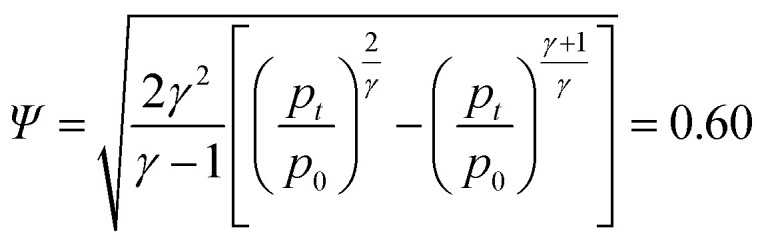


With the universal gas constant *R* = 8.314 mol J^−1^ s^−1^ and the characteristic venting rate of *ṅ*^ch^_V2_ = 1.39 mol s^−1^ the mass flux equation for an isentropic flow gives the minimal cross section of the vent-port:8
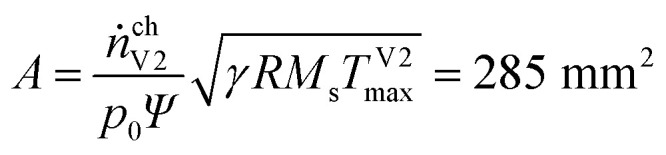
and, finally, the diameter of the vent-port:9
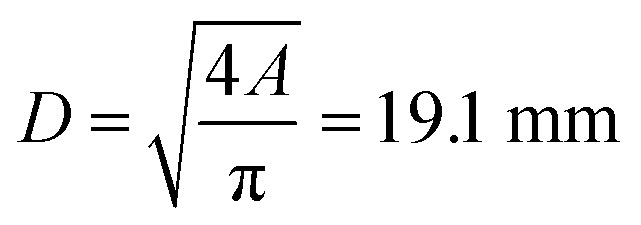


This simple calculation shows the main dependencies between the vent gas temperature, composition and venting rate on the one side and the required size of the vent port on the other side. It does not account for complex geometric factors of the vent port inlet, friction effects and heat exchange between the vent gas and battery components. Some of those effects are covered in ISO 4126.

The calculation also does not account for a possible combustion of the vent gases inside the pack. Combustion (deflagration) with air volume inside the pack would cause significant pressure and temperature increase in the pack-casing.^11^

## 7 Conclusions

Eleven overtemperature experiments were performed with prismatic metal-can Li-ion cells with a capacity of 50 A h which were produced in 2009. The experiments were done inside a custom build gas-tight heatable reactor in nitrogen atmosphere. We used four different methods to heat the cells: temperature ramp with heating of both cell sides, temperature ramp with heating of one side, stepwise heating and heating of the whole reactor.

With increasing temperature, the cells experience a sequence of events eventually finalizing in a exothermic thermal runaway reaction. An important finding is that sometimes the sequence of events is interrupted by an unforeseeable internal short circuit. When it occurs, the cell skips the remaining events and takes a direct route into final exothermic reaction. We suspect that the short circuits were caused by internal mechanical failures which damaged the electrical insulation inside the heated cells.

For cells with complex internal construction such as the tested metal-case cells it is important to repeat several test runs in order to capture the worst case outcome. The variability may be less an issue for cells with simpler construction such as pouch-type cells. The characteristics of the final reaction are: the final thermal runaway may start when the hottest point of the cell exceeds 206 °C, during thermal runaway the cell casing reaches a maximum temperature up to 594 °C and the cell releases an overall amount of up to 5.9 mol of vent-gas with up to 530 °C. When the vent-gas cools down to room temperature then part of it condensates and the remaining gas phase shrinks to a value between 2.9 mol and 4.6 mol.

Further findings are:

• The final thermal runaway reactions is triggered by either increasingly exothermic chemical reactions (classified into group A2) or by joule heat from internal short circuits (classified into group B1 or B2).

• Most cells belonged to group A2. They showed a preceding minor venting event (opening of burst plate and overpressure release from the cell) and – after further heating – a gradual onset of exothermic reactions and thermal runaway with a second major venting.

• In three experiments (group B1) internal short circuits occurred. The shorts triggered the final thermal runaway reaction and gas release before the first minor venting would normally happen.

• In one experiment (group B2) an internal short circuit triggered the thermal runaway after the first minor venting and further heating by the sample holder.

• For the tested cell type it is difficult to anticipate an imminent short circuit of a cell, because it can happen at temperatures as low as 155 °C and without previous venting, cell voltage drop or accelerating temperature increase of the cell casing.

• The cells release significant amounts of gas during thermal runaway: up to *n*_v_ = 5.95 mol (in liter at STP conditions *n*_v_ = 133 L).

• The gas release rate shows large variation from experiment to experiment, therefore several test are required for a reliable result. Up to 1.39 mol s^−1^ were measured.

• From the measured gas release rate we estimate a venting port with a diameter of at least 19.1 mm.

• The cell temperature measurements can be used to develop dynamic and spatially resolved thermal runaway models. Such models would be useful to determine at which conditions thermal runaway propagation occurs and to evaluate the effectiveness countermeasures against propagation.

In the next step we focus on newer cells with higher energy density and much higher maximal temperatures during thermal runaway. FTIR and GC equipment will be added to the test bench to quantify the vent gas composition.

## Conflicts of interest

There are no conflicts to declare.

## Supplementary Material
